# Associations between frailty and mild cognitive impairment in older adults: Evidence from rural Chiang Mai Province

**DOI:** 10.1371/journal.pone.0300264

**Published:** 2024-04-18

**Authors:** Amornphat Kitro, Jinjuta Panumasvivat, Wachiranun Sirikul, Tanasit Wijitraphan, Tharnthip Promkutkao, Ratana Sapbamrer

**Affiliations:** 1 Department of Community Medicine, Faculty of Medicine, Chiang Mai University, Chiang Mai, Thailand; 2 Environmental and Occupational Medicine Excellence Center, Faculty of Medicine, Chiang Mai University, Chiang Mai, Thailand; 3 Center of Data Analytics and Knowledge Synthesis for Health Care, Chiang Mai University, Chiang Mai, Thailand; 4 Sanpatong Hospital, Chiang Mai, Thailand; Ehime University Graduate School of Medicine, JAPAN

## Abstract

Thailand entered an aged society phase in 2000, with mild cognitive impairment (MCI) and frailty becoming prevalent among the older adult population. However, no studies have yet examined these issues specifically within rural communities. This study aims to explore the relationship between frailty and MCI among older adults in rural Thailand. It was a cross-sectional study conducted between December 2022 and June 2023. A questionnaire was administered by trained village health volunteers. The survey targeted older adults aged 60 years and above, residing in rural Chiang Mai, Thailand, with those having a history of dementia, depression, and brain injury being excluded from participation. Nine hundred eighty-four participants among the older adults were available for analysis. The mean age was 69.8 (SD 7.9) with 62.2% females (n = 612). The median frequency of exercise was three days (0–7). The prevalence of MCI and frailty among rural older adults in the community was 35.6% (n = 350) and 8% (n = 79), respectively. There were four factors associated with an increased risk of MCI, including age (aOR = 1.07, 95% CI 1.04–1.09, p < 0.001), smoking cigarettes (aOR 1.95, 95% CI 1.27–2.98, p = 0.002), feelings of loneliness (aOR 1.43, 95% CI 1.01–2.03, p = 0.043), and the presence of frailty (aOR 1.92, 95% CI 1.10–3.35, p = 0.022). There were two factors associated with a lower risk of MCI: a higher education level (aOR 0.90, 95% CI 0.86–0.94, p <0.001) and engaging in frequent exercise (aOR 0.9, 95% CI 0.86–0.95, p < 0.001). Frailty exhibited an association with an elevated risk of MCI among older adults in rural communities. Enhancing screening through health volunteers and primary healthcare professionals, coupled with bolstering community-driven health promotion initiatives, becomes imperative.

## Introduction

Thailand has grappled with the challenges of an aging society since 2000, where individuals aged 60 and above constitute over 10% of the Thai population [1]. Currently, the Thai older adult demographic accounts for 18.3% (12.1 million) of the total population. This comprises early older adults (60–69 years old) at 56.5% (6.8 million), mid-older adults (70–79 years old) at 29.1% (3.5 million), and late older adults (80 years old and above) at 14.4% (1.8 million) [2]. Chiang Mai, the second largest province, reflects this trend with 3% of its population classified as older adults, constituting 0.3 million individuals, and witnessing a steady increase [2]. The older adult demographic emerges as a vulnerable segment, facing challenges in both physical and mental health due to the inevitable effects of aging, particularly cognitive impairment [3]. This impairment can result in a decline in their daily functional capacities, affecting not only individuals but also families, communities, and even the broader Thai economy [3]. The vulnerability is particularly pronounced in rural areas, characterized by non-urban settings with fewer than 50,000 residents and limited access to formal care services, typically supported by only one or two generalist physicians, along with a nurse practitioner or physician assistant [4]. This urban-rural disparity also intensifies the impact on the health and well-being of older adults residing in rural communities, compounding the challenges of poorer access to necessary formal care services [5].

Mild cognitive impairment (MCI) represents the highest-risk condition leading to early dementia. Problems with forgetfulness may progress to dementia within one to three years, with a mortality rate about two times higher than that of the general older adult population [6, 7]. In Thailand, the prevalence of MCI among the urban population was 52.8%, surpassing the global average prevalence of 6.7–25.2% [8, 9]. Older adults with MCI typically experience impairment across five dimensions of the brain including memory, language, visual-spatial skill, attention, and executive function [10]. Several factors were associated with an increased risk of MCI in older adults. These include an age increase (1.1 to 1.6 times), a history of smoking (1.2 times), the presence of chronic medical conditions like diabetes, hypertension, and dyslipidemia (4.4 times), as well as anxiety disorders (2.2 times) [8, 11–16]. Additionally, heightened risk was observed in cases of polypharmacy and diminished physical activity [8, 11–16].

Frailty emerges as a prevalent geriatric syndrome, arising from physiological changes linked to the aging process. This transformation contributes to a decline in both physical and mental well-being, resulting in diminished quality of life, heightened mortality rates, and a demand for intricate and advanced care [17]. The indicators of frailty are intertwined with age-related degeneration, including muscle fatigue, reduced physical activity, slowed gait, unintentional weight loss, and increased vulnerability across physical, mental, and social domains. This results in compromised adaptability, disrupted homeostasis and heightened susceptibility to illness [18–21]. A recent systematic review revealed that frailty was 1.8 times (95% CI 1.1–2.9) associated with MCI while Asians such as Japanese and Singaporeans had a 6.4 times higher chance for MCI [16, 22, 23]. Participants who exhibited both frailty and MCI faced a higher mortality rate [16, 22, 24]. The presence of either MCI or frailty, or their combination, has the potential to significantly diminish the quality of life, escalate vulnerability, heighten the need for permanent caregiving, and increase the demand for healthcare assistance, ultimately leading to elevated mortality among community-dwelling older adults. However, there is currently insufficient evidence to establish a clear connection in the rural population [25, 26].

Currently, a substantial gap exists in the comprehension of frailty and MCI among older adults residing in rural regions of Thailand. The primary objectives of this study involve exploring the association between frailty and MCI and examining the risk factors linked to MCI among older adults in the rural areas of Chiang Mai Province. Consequently, investigating this knowledge will serve as a pivotal tool for identifying high-risk groups within the older adult population in rural communities. This endeavor aims to proactively prevent the progression of cognitive impairment or dementia among older adults in the future.

## Materials and methods

### Study design and population

The study employed a cross-sectional research design. Utilizing a convenient sampling method. It was conducted among the older adult population residing in a rural community in Chiang Mai, Thailand. The investigation period spanned 1 December 2022 and 30 June 2023. Inclusion criteria consisted of individuals aged 60 years or older, currently living in the rural areas of Chiang Mai, proficient in Thai language communication, and willing to provide written consent for participation with research assistants trained and supervised by the project leader. The recruitment process was initiated by contacting health-promoting hospitals in rural areas in Chiang Mai and providing research information to all older adults residing in the rural community. Upon expressing willingness to participate in the study, eligible participants were asked to provide their signature on the informed consent form. Participants were then given the option to independently complete the questionnaire or receive assistance from research personnel. Older adult individuals with dementia, depression, and a history of brain injury were excluded from the study.

### Questionnaire

Survey questionnaires were used to collect data from the older adults who met the eligibility criteria. The questionnaire was made up of sociodemographic and health information.

1. sociodemographic information: age (year), gender (male/female), years of education (year), marital status (single/marriage/separate), living status, working status (working/no job or retired), monthly salary (< 26,000, ≥ 26,000 Baht/month), having caregiver (yes/no), and feeling loneliness (yes/no).
2. Health information: medical conditions (yes/no), medication use, alcohol consumption (yes/no), active cigarette smoking (yes/no), frequency of exercise (days/week), history of hospital admission in the past year (yes/no), history of falling in the past year (yes/no), sleep quality (good/poor), and health perception (good/poor)

### Measures

The measurement process involved the utilization of three tools.

#### The Thai Mini-Mental State Examination (TMSE)

It was employed to evaluate the cognitive function. Out of the 30-item questionnaire, a score below 24 was indicative of mild cognitive impairment [27, 28].

#### Thai FRAIL scale

The Thai FRAIL scale consists of five self-reported questions: Do you experience fatigue? Can you climb a flight of stairs? Can you walk a block without stopping? Are you being treated for more than five medical conditions? Have you experienced an unintentional weight loss of 5% or more of your body weight in the last six months”. The total score ranges from 0 to 5 points and those with a score of 3 to 5 points are categorized as "frail." [29].

#### Thai PHQ-9

The Thai PHQ-9 consisted of 9 questions that were used to determine the severity of depression during the preceding two weeks. Each item in the PHQ-9 was rated on a 0–3 scale (0 = never, 1 = several days (1–7 days), 2 = more than a week (> 7 days), 3 = every day). The comprehensive PHQ-9 score varies from 0 to 27, and it is categorized into four groups: mild depression (score 5–9), moderate depression (score 10–14), moderately severe depression (score 15–19), and severe depression (score 20 or more) [30].

### Statistical analysis

The statistical analysis was performed using Jamovi. Descriptive statistics, including frequency (n), percentage (%), mean, median, standard deviation (SD.), the 25^th^ percentile (P^25th^), and the 75^th^ percentile (P^75th^) were used. Univariable analysis, including independent t-test, Mann-Whitney U test, and Fisher’s Exact test was used to investigate the variables associated with mild cognitive impairment. The variables that had a significant (p < 0.05) from the univariable analysis were included in the multivariate model. Binary logistic regression analyses were used to investigate the factors associated with health symptoms. Adjusted odds ratio (adj. OR) and 95% confidence intervals (95% CI) were presented.

### Ethics consideration

This study was approved by the Research Ethics Committee of the Faculty of Medicine, Chiang Mai University (Research Project Certification Document No. COM2565-09302).

## Results

This survey gathered 984 participants from older adult individuals residing in the community of Chiang Mai, Thailand. Among individuals identified with MCI was 35.5% (n = 350), and the mean age was 72.7 years (SD = 8.8). 60.9% (n = 213) were female. The median year of education was four years (IQR 0–5). 68.0% (n = 238) were married, 9.4% (n = 33) were living alone and 71.2% (n = 249) were retired or currently unemployed. 82.8% (n = 288) had chronic medical conditions and were taking an average of two medications (IQR 0–3). 33.7% (n = 118) were alcohol consumption and 16% (n = 56) were smoking. 71.1% (n = 249) had a caregiver and 80.9% (n = 283) reported a monthly salary below 26,000 THB (750 USD). Most of this group engaged in exercise two times a week (IQR 0–7). 32.0% (n = 112) reported feeling lonely. More than half (25.1%) were admitted to the hospital in the past year, and 28.3% (n = 99) had experienced falls during the last year. The majority of them slept for around eight hours, but around 39.7% (n = 139) had poor sleep quality. 45.1% (n = 158) considered their health condition to be poor and 27.4% (n = 96) believed their cognitive function to be poor as well. Participants had 8% frailty (n = 79), mild depression 6.5% (n = 64), and moderate to severe depression 1.1% (n = 11) (Table 1).

**Table 1:** Socio-demographic characteristics of older adults living in rural areas in Chiang Mai.

Characteristics	Total N (%)	Mild cognitive impairment		*P*-value
		Yes (350, 35.5%)	No (634, 64.5%)	
Age (years.), mean+SD.	69.8±7.9	72.7±8.8	68.2±6.8	<0.001
Gender				
Female	612 (62.2)	213 (60.9)	399 (62.9)	0.537
Male	372 (37.8)	27 (34.2)	345 (38.1)	
Years of education (years), median (IQR)	4 (4,6)	4 (0, 5)	4 (4,6)	<0.001
Marital status				
Married	674 (58.5)	238 (68.0)	436 (68.8)	0.830
Others	310 (31.5)	31 (39.2)	279 (30.8)	
Living status				
Living alone	878 (89.2)	33 (9.4)	73 (11.5)	0.335
Living with others	106 (10.8)	8 (10.1)	98 (10.8)	
Working status				
Working	272 (27.6)	101 (28.9)	171 (27.0)	0.001
No job/retired	712 (72.4)	249 (71.2)	463 (73.6)	
Chronic medical conditions	774 (79.9)	288 (82.8)	486 (78.3)	0.096
Numbers of medication use (types/day), median (IQR)	2 (0,3)	2 (0, 3)	1 (0, 3)	0.026
Alcohol consumption	369 (37.5)	118 (33.7)	251 (39.6)	0.074
Cigarette smoking (active)	117 (11.9)	56 (16.0)	61 (9.6)	0.004
Having a caregiver	665 (67.6)	249 (71.1)	416 (65.6)	0.088
Monthly salary				
< 26,000 Baht/month	211 (21.4)	16 (20.3)	195 (21.5)	0.196
≥ 26,000 Baht/month	773 (78.6)	67 (19.1)	144 (22.7)	
Frequency of exercise (days/week) median (IQR)	3 (0,7)	2 (0, 7)	4 (1, 7)	<0.001
Feel loneliness	223 (22.7)	112 (32.0)	111 (17.5)	<0.001
History of hospital admission in the past year	212 (21.5)	88 (25.1)	124 (19.6)	0.043
History of falling in the past year	218 (22.2)	99 (28.3)	119 (18.8)	0.001
Duration of sleeping (hours.), mean+SD.	7.9 (7.9,8)	7.9+0.9	7.9+0.9	0.677
Sleep Quality				
Poor quality	360 (36.6)	139 (39.7)	221 (34.9)	0.147
Good quality	624 (63.4)	211 (60.3)	413 (65.1)	
What do you think about your health?				
Poor health	393 (39.9)	158 (45.1)	235 (37.1)	0.014
Good health	591 (60.1)	192 (54.9)	399 (62.9)	
What do you think about your cognitive abilities?				
Poor cognitive	270 (27.4)	96 (27.4)	174 (27.4)	0.996
Good cognitive	714 (72.6)	254 (72.6)	460 (72.6)	
Frailty	79 (8.0)	49 (14.0)	30 (4.7)	<0.001
Depression				
Very mild	909 (92.4)	306 (87.4)	603 (95.1)	<0.001
Mild	64 (6.5)	36 (10.3)	28 (4.4)	
Moderate	6 (0.6)	4 (1.1)	2 (0.3)	
Severe	5 (0.5)	4 (1.1)	1 (0.2)	

SD, Standard Deviation; IQR, Interquartile range

The unadjusted odds ratio (OR) for the relationship between frailty and MCI in older adults living in rural areas of Chiang Mai, Thailand, was 3.28 (95% CI = 2.038, 5.270, p- < 0.001). Table 2 presents the factors associated with MCI among older adults residing in rural areas of Chiang Mai, Thailand. There were four factors associated with an increased risk of MCI including age (aOR = 1.07, 95% CI 1.04–1.09, p < 0.001), cigarette smoking (aOR 1.95, 95% CI 1.27–2.98, p = 0.002), feelings of loneliness (aOR 1.43, 95% CI 1.01–2.03, p = 0.043), and presence of frailty (aOR 1.92, 95% CI 1.10–3.35, p = 0.022). There were two factors associated with a lower risk of MCI including higher education level (aOR 0.90, 95% CI 0.86–0.94, p < 0.001) and engaging in frequent exercise (aOR 0.9, 95% CI 0.86–0.95, p < 0.001) (Table 2).

**Table 2:** Factors associated with mild cognitive impairment among older adults living in rural areas in Chiang Mai.

Factors	Adjusted OR	95% CI	*P*-value
Frailty	1.916	1.097, 3.345	0.022
Age	1.065	1.044, 1.086	<0.001
Years of education	0.901	0.861,0.944	<0.001
Working status	0.820	0.598, 1.125	0.219
Cigarette smoking	1.945	1.271, 2.977	0.002
Number of drug types	1.033	0.943, 1.132	0.481
Frequency of exercise	0.900	0.857, 0.945	<0.001
Feel loneliness	1.432	1.012, 2.028	0.043
Falling in the past one year	1.060	0.740, 1.519	0.750
Admit to hospital in the past year	0.921	0.641, 1.322	0.654
Health perception- poor health	0.931	0.681, 1.272	0.652
Depression, (Very mild as ref.)			
Mild	1.660	0.917, 3.006	0.094
Moderate	3.199	0.475, 21.550	0.232
Severe	7.093	0.697, 72.195	0.098

CI, confident interval; OR, Odds ratio.

## Discussion

To our knowledge, this was the first study that represented the association between frailty and MCI within rural communities in Thailand. Among the older adult population residing in these rural areas, the prevalence of MCI was recorded at 35.6%. Several factors emerged as significant contributors to the elevated risk of MCI, encompassing age, current smoking, and feelings of loneliness. Conversely, engaging in regular exercise and having a high education level were associated with a reduced risk of MCI among the older adult inhabitants of these rural communities.

Our study findings demonstrated an association between frailty and MCI among older adults residing in rural communities, showing an elevated risk of MCI by 1.9 times (95% CI 1.1–3.5; p 0.02) for those with frailty. These results align with a systematic review and meta-analysis conducted in 2015 and 2019, which similarly reported a 1.8-fold increase in the risk of cognitive impairment (95% CI = 1.11–2.92; p = 0.02), with severity influenced by higher levels of frailty [22, 24]. Our research revealed a greater association compared to prior studies. Furthermore, a study conducted in rural India also provides backing for the correlation between frailty and cognitive impairment [31]. This suggests that older individuals residing in rural areas may possess a potential for developing age-related conditions such as frailty accompanied by cognitive impairment.

Our study revealed the highest documented prevalence of MCI, reaching 35.6%, among older adults residing in rural communities. This prevalence surpassed rates observed in rural Taiwanese older adults (25.1%) and in urban older adult populations of China and India, where the range spanned from 9.67% to 26.06% [14, 32–36]. This can be attributed to the fact that rural areas often exhibit lower levels of education, limited access to healthcare services and resources, lower socioeconomic status, and occupations with fewer skill requirements. These factors may potentially restrict the accumulation of cognitive reserve, contributing to a decline in brain function [32, 37, 38]. Additionally, residents in rural areas often experience delays in the screening and inadequate management of chronic diseases [32, 37]. In comparison to the urban Thai population, the occurrence was lower (35.6% vs. 52.8%). This discrepancy in prevalence can be clarified by variations in assessment tools. It likely stemmed from a selection bias, where urban environments tend to encompass a higher number of cases involving multiple medical conditions compared to community settings, sociodemographic factors, and living conditions.

Our study findings indicate that regular physical exercise among older adults was associated with a 14% reduction in the risk of MCI (95% CI 0.857, 0.945; p < 0.001). This outcome aligns with investigations conducted by Smith et al. and Li et al. in various countries, including China, Ghana, India, Mexico, Russia, and South Africa. These studies demonstrated that greater engagement in physical activities or maintaining high levels of physical activity was linked to a decreased risk of MCI [12, 34, 36]. Moreover, one study conducted among older adults living in rural northern China demonstrated that engaging in physical activity was a protective factor for MCI (p < 0.05), and the saturation point was identified at 6546 MET x min/week [39]. Engaging in aerobic or resistance exercises among older adults can enhance cardiovascular function, provide greater nourishment to brain tissue cells, preserve brain functionality, enhance memory, and postpone the progression of neurodegenerative processes [40, 41].

It is recommended that Thai older adult populations living in rural communities undergo early frailty screening with T-FRAIL and cognitive screening with TMSE [27–29]. Community health volunteers and primary care physicians could play a pivotal role in administering that screening tool to identify older adults at risk. To mitigate this prevalence in Thailand, the establishment of elder-friendly community centers (schools for elders) and senior citizen clubs could serve as an engaging solution. Those centers could gather older adults from the community, enhancing physical and mental well-being. The program should aim for a minimum of 150 minutes of moderate aerobic physical activity weekly, supplemented by resistance training or multifaceted exercise. Moreover, it holds the potential to alleviate feelings of loneliness among older adults living alone, consequently reducing the risk of later cognitive impairment. A study conducted in Singapore lends support to this notion, indicating that engaging in social activities during later life can reduce the risk of MCI by approximately 57% [42, 43]. This comprehensive approach targets modifiable risk factors for cognitive decline. The success of such a program could be tailored through collaborative efforts involving doctors and physical therapists within each respective catchment area [44, 45].

Our study had a few limitations, primarily relying on a convenient sampling of older adults within rural communities. Additionally, the cross-sectional design of the study prevented the establishment of causal relationships between frailty and MCI among these older adults. Loneliness was subjectively evaluated without the use of comprehensive tools. To overcome these limitations, future research should encompass multiple sites across Thailand, employing proper screening tools in a prospective cohort design that compares urban and rural settings. This approach would allow for a comprehensive overview, guiding policymakers and stakeholders in advocating for the implementation of screening programs and the development of health promotion initiatives for older adults in rural communities. Such efforts would contribute to empowering these older adults, enabling them to have self-efficacy in sustaining their competence and decreasing potential modifiable risks that could lead to cognitive impairment in the foreseeable future.

## Conclusion

Approximately 35.6% of the senior population residing in rural areas experience MCI. The likelihood of developing MCI is heightened among older adults who are frail or experience feelings of loneliness. However, the risk can be mitigated by fostering increased physical activity. For those older adult individuals at a higher risk, it is recommended to employ accessible screening methods like the TMSE to identify cognitive impairment and the T-FRAIL assessment for identifying frailty. These tools can effectively detect issues early on, preventing the progression towards irreversible disabilities. To address this concern, supporting community initiatives like older adult schools and senior citizen clubs becomes crucial. By establishing health promotion programs within these contexts, we can encourage greater participation in engaging activities among older adults and promote enhanced physical activity levels, especially among those residing in rural settings.

## Supporting information

S1 File Data regarding case summaries. (XLSX)
